# #MadelungDeformity: Insights Into a Rare Congenital Difference Using Social Media

**DOI:** 10.1177/15589447211054133

**Published:** 2021-11-12

**Authors:** Abbas Peymani, Max M. Lokhorst, Austin D. Chen, Chantal M.A.M. van der Horst, Bernard T. Lee, Samuel J. Lin, Simon D. Strackee

**Affiliations:** 1 University of Amsterdam, The Netherlands; 2 Harvard Medical School, Boston, MA, USA

**Keywords:** congenital hand, hand surgery, Madelung deformity, research and health outcomes, social media

## Abstract

**Background:**

Madelung deformity is a rare congenital hand difference with little known regarding the patient perspective. In this cross-sectional survey study, we harnessed the global reach of social media to understand the clinical spectrum of Madelung deformity and its impact on physical, mental, and social health.

**Methods:**

A survey was developed based on a previously published protocol and multiple Patient-Reported Outcomes Measurement Information System (PROMIS) short forms. The survey was distributed on several Madelung deformity communities on Facebook and Instagram. *T*-scores were calculated, interpreted, and compared between patients who underwent surgery and those who did not. Correlations between scores were calculated using the Spearman rank correlation coefficient.

**Results:**

Mean PROMIS scores for adults were as follows: pain intensity, 4.9 ± 2.8; pain interference, 57.6 ± 10.0; upper extremity, 35.2 ± 8.1; depression, 53.8 ± 11.1; anxiety, 55.4 ± 11.4; and ability to participate in social roles and activities, 42.5 ± 7.7. Mean scores for children were as follows: pain intensity, 5.0 ± 2.8; pain interference, 55.7 ± 11.3; upper extremity function, 24.6 ± 10.4; depressive symptoms, 57.7 ± 11.3; anxiety, 57.3 ± 11.9; and peer relationships, 42.2 ± 10.3.

**Conclusions:**

Madelung deformity has significant effects on patients’ physical, mental, and social well-being, even after surgical treatment. Using social media, we were able to compensate for Madelung deformity’s rarity by engaging an international audience, demonstrating the feasibility to conduct research through it, and providing a global perspective of the disease entity.

## Introduction

In April 1878, after a lecture in Berlin by a German surgeon, the condition “Madelung deformity” was introduced to the surgical community.^1^ This congenital hand difference is caused by a premature growth arrest of the distal radius, leading to a shortened radius with a volar and ulnar angulation, relative overgrowth of the ulna, and pyramidization of the proximal carpal row.^2^ The deformity is often caused by mutations in the short stature homeobox (SHOX) gene, which is associated with both Leri-Weill dyschondrosteosis and Turner syndrome.^3,4^ Madelung deformity is extremely rare, and its incidence and prevalence are relatively unknown, with hand surgeons seeing very few, if any, cases during their surgical career.^5^ This is evident by the sparse literature, with the largest recently published case series study including 19 patients,^6^ raising several unanswered questions regarding etiology, diagnostics, classifications, treatment options, and surgical outcomes.^7^ Furthermore, small-powered studies often inadequately describe functional status, and only a handful of studies have even considered the patient perspective.^8^

This is remarkable, given that it is well established that congenital hand differences have a profound and lifelong impact on the physical, mental, and social aspects of patients’ lives.^9^ To increase our understanding of these deformities, it is therefore paramount to perform studies using validated patient-reported outcome measures (PROMs) in a large sample size of patients.^10^ Although the investigation of PROMs through combining data from multiple centers has provided us insights into other congenital hand differences,^11^ this has not translated to an increased understanding of Madelung deformity due to the low numbers of patients captured.^12^ To date, a potentially useful tool to study these rare patient populations that may otherwise be extremely difficult to reach is social media.^13^

This study’s primary aim is to understand the clinical spectrum of Madelung deformity and its impact on physical, mental, and social health by harnessing the global reach of social media. Using the universal Patient-Reported Outcomes Measurement Information System (PROMIS),^14^ advised for use in rare diseases,^15,16^ we collaborated with several social media communities to conduct a cross-sectional survey. The PROMIS allows for an assessment of multiple physical, mental, and social health domains and has been proven to be efficient and reliable for different upper extremity conditions.^17^ To the best of our knowledge, this is the first and largest-to-date study of PROMs for Madelung deformity.

## Materials and Methods

### Study Population and Data Collection

**Figure 1.**
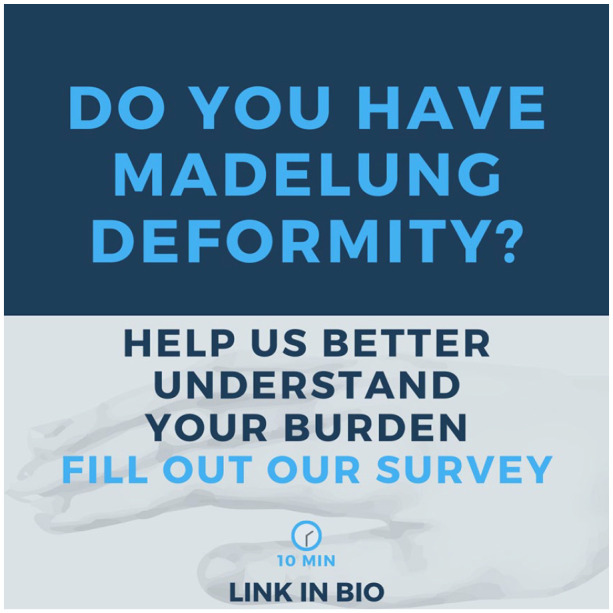
Example post on the social media platform Instagram.

**Figure 2.**
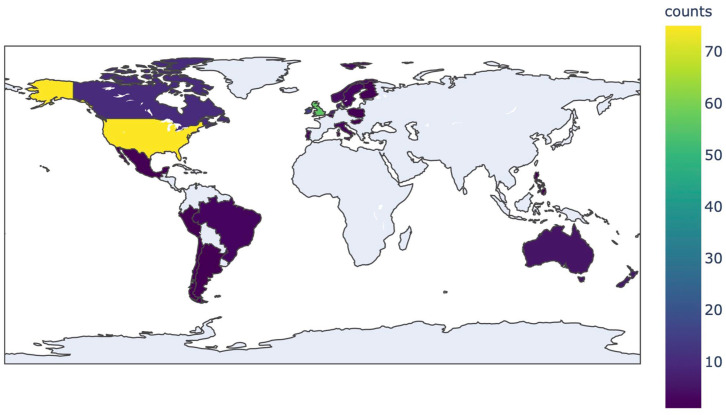
Heatmap showing initial engagement from social media.

Ethical approval for this cross-sectional survey study was waived by our hospital Medical Ethics Committee. Data were collected through an online survey created with Google Forms. The survey was distributed through a post (Figure 1) on existing online Madelung deformity communities on Facebook and an Instagram community established by the authors (“@madelungdeformity”). The survey was kept online for a total of 9 days from the initial launch date (February 11, 2020). Figure 2 shows an overview of the geographic locations of all persons who visited the link (n = 207). The exact location of participants who fully completed the survey was not available due to privacy reasons. The survey consisted of questions on demographic characteristics and PROMIS short forms.^14^

### Survey: Demographic Characteristics

Questions regarding demographic characteristics were based on a previously published protocol that proposed various changes in the methodology of future Madelung deformity studies.^8^ The following variables were collected: age, assigned sex at birth, height, weight, affected arm(s), previously diagnosed genetic conditions, previously diagnosed medical conditions, medication use (including painkillers), family history of Madelung deformity, and any (corrective) surgeries of the hand, wrist, or arm. Based on the selected age range (18+ or 8-17 years), participants were presented with either adult or pediatric versions of the PROMIS forms. The complete question list is available in Supplementary File 1.

### Survey: PROMIS Short Forms

The PROMIS short forms consisted of fixed sets of questions for 6 health domains,^14^ covering physical, mental and social health. For adults, the following forms and versions were used: (1) Pain Intensity 1a Version 1.0; (2) Pain Interference 8a Version 1.0; (3) Upper Extremity 7a Version 2.0; (4) Depression 8a Version 1.0; (5) Anxiety 8a Version 1.0; and (6) Ability to Participate in Social Roles and Activities 8a Version 2.0. For children, we used the short forms: (1) Pediatric Pain Intensity 1a Version 1.0; (2) Pediatric Pain Interference 8a Version 2.0; (3) Pediatric Upper Extremity 8a Version 2.0; (4) Pediatric Depressive Symptoms 8a Version 2.0; (5) Pediatric Anxiety 8a Version 2.0, and (6) Pediatric Peer Relationships 8a Version 2.0; Regarding pain, “Pain Intensity” assesses how much a person hurts, whereas “Pain Interference” considers self-reported consequences of pain on relevant aspects of one’s life.

### Postprocessing and Data Analysis

After exporting raw survey data from Google Forms, each entry was manually inspected to remove errors (eg, previous surgeries not involving the upper extremity), perform metric unit conversions (eg, pounds to kilograms for weight), and determine analgesic use by reviewing all listed medications. The PROMIS short forms were scored using a *T*-score metric, in which 50 is the mean and 10 is the standard deviation of a relevant reference population.^18^ *T*-scores were calculated based on scoring tables of each domain’s published manuals, using self-developed software in Python (Python Version 3.7.4). Higher scores represent a higher level of the measured domain,^14^ and their interpretation in different domains^19^ is shown in Supplementary Figure 1.

Means were compared between patients who underwent surgery and those who did not. For normally distributed scores, an independent-samples *t* test (variances equal) or Welch *t* test (variances not equal) was performed. Equality of variances was assessed using the Levene test. For nonnormally distributed scores, the Mann-Whitney *U* test was performed. All analyses were performed separately for adult and pediatric patients. Correlations between the different PROMIS short form scores were calculated using the Spearman rank correlation coefficient (ρ), with correlation strength interpreted as low (<0.3), moderate (0.3-0.5), or high (>0.5).

## Results

### Patient Characteristics

An overview of the patient characteristics is shown in Table 1. Of the 207 persons who opened the survey, 133 (64%) completed it. Participants’ mean age was 34.8 ± 12.5 years, with 116 adults and 17 children. Nearly all participants (99%) were assigned female sex at birth. A total of 55 participants (41%) reported that they had undergone previous surgical correction of the wrist with a mean age of 20.5 ± 9.5 years at their first surgery and a mean of 2.4 ± 2.7 surgeries in total per participant. Most participants (92%) reported the deformity to occur bilaterally.

**Table 1.** Patient Characteristics.

Variable	Madelung deformity (n = 133)	Adult (n = 116)	Children (n = 17)
Age, y	34.8 ± 12.5	37.6 ± 10.8	15.6 ± 2.8
Age at diagnosis, y	19.4 ± 11.3	20.5 ± 11.6	11.7 ± 2.4
Female	132 (99%)	115 (99%)	17 (100%)
Height, cm	156.9 ± 9.7	157.0 ± 9.6	156.2 ± 10.4
Weight, kg	71.1 ± 21.1	73.7 ± 21.1	52.8 ± 8.8
Body mass index	28.8 ± 8.3	29.9 ± 8.3	21.6 ± 2.6
Right hand dominance	118 (89%)	102 (88%)	16 (94%)
Bilateral deformity	123 (92%)	107 (92%)	16 (94%)
Familiar history	62 (47%)	58 (50%)	4 (24%)
Confirmed genetic mutation	37 (28%)	32 (28%)	5 (29%)
Analgesic use	65 (49%)	57 (49%)	8 (47%)
Underwent surgery	55 (41%)	45 (39%)	10 (59%)
Age at first surgery, y	20.5 ± 9.5	21.8 ± 9.9	14.6 ± 4.3
Mean number of surgeries	2.4 ± 2.7	2.4 ± 2.9	2.1 ± 1.4

### PROMIS Outcomes

Descriptive data of the PROMIS short forms are presented for adults and children in Tables 2 and 3, respectively. Mean PROMIS scores for adults were as follows: pain intensity, 4.9 ± 2.8; pain interference, 57.6 ± 10.0; upper extremity, 35.2 ± 8.1; depression, 53.8 ± 11.1; anxiety, 55.4 ± 11.4; and ability to participate in social roles and activities, 42.5 ± 7.7. No significant differences were found between patients who underwent surgery and those who did not. Mean scores for children were as follows: pain intensity, 5.0 ± 2.8; pain interference, 55.7 ± 11.3; upper extremity function, 24.6 ± 10.4; depressive symptoms, 57.7 ± 11.3; anxiety, 57.3 ± 11.9; and peer relationships, 42.2 ± 10.3. A significantly lower level of pain interference was seen in pediatric patients who underwent surgery (51.0 ± 12.0 vs 62.3 ± 5.2; *P* = .045).

**Table 2.** Mean PROMIS Scores in Adults With Madelung Deformity.

Domain	Madelung deformity (n = 116)	Unoperated patients (n = 71)	Operated patients (n = 45)	*P* value
Pain intensity	4.9 ± 2.8	4.7 ± 2.8	5.0 ± 2.9	.567
Pain interference	57.6 ± 10.0	57.2 ± 10.5	58.3 ± 9.1	.650
Upper extremity	35.2 ± 8.1	36.2 ± 8.2	33.5 ± 7.6	.081
Depression	53.8 ± 11.1	53.5 ± 10.5	54.3 ± 12.1	.692
Anxiety	55.4 ± 11.4	54.9 ± 11.3	56.1 ± 11.4	.733
Social^a^	42.5 ± 7.7	43.0 ± 7.6	41.8 ± 7.8	.455

*Note*. PROMIS = Patient-Reported Outcomes Measurement Information System.

a Ability to participate in social roles and activities.

**Table 3.** Mean PROMIS Scores in Children With Madelung Deformity.

Domain	Madelung deformity (n = 17)	Unoperated patients (n = 7)	Operated patients (n = 10)	*P* value
Pain intensity	5.0 ± 2.8	6.3 ± 2.2	4.1 ± 2.8	.129
Pain interference	55.7 ± 11.3	62.3 ± 5.2	51.0 ± 12.0	.045
Upper extremity function	24.6 ± 10.4	21.7 ± 8.2	26.7 ± 11.3	.363
Depressive symptoms	57.7 ± 11.3	57.9 ± 11.6	57.5 ± 11.2	.943
Anxiety	57.3 ± 11.9	59.3 ± 14.0	56.0 ± 10.0	.607
Peer relationships	42.2 ± 10.3	42.1 ± 5.7	42.3 ± 12.5	.969

*Note*. PROMIS = Patient-Reported Outcomes Measurement Information System.

### Correlations Between PROMIS Short Forms

Correlations between the PROMIS short form scores are shown in Table 4 for adults and Table 5 for children. Notably, upper extremity function showed an inversely high correlation with pain intensity (ρ = −0.67), pain interference (ρ = −0.70), and depression (ρ = −0.54) in adults and with pain intensity (ρ = −0.64) and pain interference (ρ = −0.66) in children.

**Table 4.** Spearman Rank Correlation for PROMIS Scores in Adults With Madelung Deformity.

Domain	Pain intensity	Pain interference	Upper extremity function	Depression	Anxiety	Social^a^
Pain intensity	—	0.80	−0.67	0.37	0.32	−0.74
Pain interference	0.80	—	−0.70	0.46	0.46	−0.81
Upper extremity function	−0.67	−0.70	—	−0.54	−0.48	0.80
Depression	0.37	0.46	−0.54	—	0.76	−0.50
Anxiety	0.32	0.46	−0.48	0.76	—	−0.46
Social^a^	−0.74	−0.81	0.80	−0.50	−0.46	—

*Note*. PROMIS = Patient-Reported Outcomes Measurement Information System.

a Ability to participate in social roles and activities.

**Table 5.** Spearman Rank Correlation for PROMIS Scores in Children With Madelung Deformity.

Domain	Pain intensity	Pain interference	Upper extremity function	Depressive symptoms	Anxiety	Peer relationships
Pain intensity	—	0.79	−0.64	0.52	0.30	−0.45
Pain interference	0.79	—	−0.66	0.24	0.27	−0.17
Upper extremity function	−0.64	−0.66	—	−0.04	0.07	0.01
Depressive symptoms	0.52	0.24	−0.04	—	0.67	−0.54
Anxiety	0.30	0.27	0.07	0.67	—	−0.41
Peer relationships	−0.45	−0.17	0.01	−0.54	−0.41	—

## Discussion

This cross-sectional survey highlights Madelung deformity’s impact from the patient perspective, showing significant effects of the disease on one’s physical, mental, and social health. Even after surgical treatment, the health burden of this congenital hand difference remains. Despite Madelung deformity’s rarity, we were able to engage a relatively broad international audience through the use of social media, allowing us to assess patient outcomes and determine demographic characteristics.

The overwhelmingly female patient composition and bilateral occurrence of Madelung deformity have been extensively described^20^; however, no population-based data or epidemiological studies are available. Therefore, our knowledge regarding demographic characteristics has been derived from small-powered studies.^6^ Mean reported adult patient height in our study was 157 cm (5′2″), which is at the 27th percentile for women in the United States. Previous studies reported average heights of 159 cm or below standard heights at the 19th percentile,^2,21^ not surprising given that a substantial proportion of patients with Madelung deformity have confirmed SHOX gene mutations, which is strongly associated with short stature and dyschondrosteosis.^3,4^ Of our participants, 28% reported having this genetic mutation, and 47% reported on Madelung deformity occurring in family members. Interestingly, it has been hypothesized that a substantial amount of patients have underlying diagnoses of dyschondrosteosis.^22,23^ The collection of genetic material in future prospective studies, as recommended in a previously published protocol,^8^ will hopefully provide a definite answer.

While the often-reported pain is the primary indication for surgical treatment, its quantification in a preoperative or postoperative setting has not been consistent. Some studies measured pain using a Numeric Rating Scale (NRS) or Visual Analog Scale (VAS), reporting preoperative scores of 9/10 and 50/100, and postoperative scores of 0/10 and 22.5/100, respectively.^24,25^ One study using a “Pain Score” (0 = requiring narcotic medication, 30 = no pain) reported scores of 14.5 and 25.0 before and after surgery, respectively.^26^ Another study only reported preoperative measurements of 3.3/4 (0 = no pain, 4 = continuous pain).^6^ This widely heterogeneous registration of pain is suboptimal, and studies should quantify pain in an analogous manner using a VAS or NRS, both before and after surgery, without merging the variable in a “wrist score.”^27^ In this study, we not only recorded pain intensity but also highlighted the consequences of pain on a person’s life, showing mild interference in adults, moderate interference in children who did not undergo surgery, and mild interference in children who underwent surgery; notably, 49% of participants reported the use of analgesics. The presence of pain in surgical patients postoperatively seems in stark contrast to the literature. On the one hand, there is potential for bias in this cross-sectional survey study as symptomatic persons might be more vocal about their symptoms and engage in social media communities. On the other hand, it may be that a short follow-up period or loss of follow-up results in underreporting of this outcome in the literature, as most studies have not quantified pain or have only reported preoperative measurements.^6,8^ Nevertheless, in future studies we would be most interested in the difference between preoperative and postoperative pain instead of single cross-sectional measurements.

The Oberg-Manske-Tonkin (OMT) classification, a proven and adopted classification framework for congenital hand and upper limb anomalies,^10,28^ most recently reclassified Madelung deformity as an entire upper limb malformation of the radioulnar (anteroposterior) axis (OMT type IA2.vii).^29^ A previous study applying PROMIS to congenital hand anomalies reports median upper extremity scores of 37 (<11 years) and 45 (11-17 years) in children with entire limb malformations (OMT type IA) and worse upper extremity function in children with bilateral compared with unilateral deformities.^11^ In contrast, another study reported scores within the normal range in 41 children (5-17 years) with a similar OMT type.^30^ Our results show moderately impaired functioning in adults (35.2 ± 8.1) and severely impaired functioning in children (24.6 ± 10.4). A noteworthy point is the slightly lower postoperative score in adults and a slightly higher score in children, albeit not significant. The results of our study, which mainly involves data entries from adults with Madelung deformity (a subset of type IA malformations), can unfortunately not be compared with these aforementioned studies. However, the bilateral occurrence (92%), the lack of consensus regarding optimal treatment, and the relatively late age of onset, diagnosis, and corrective surgery might all play a role in decreased upper extremity function.^31^

Our findings suggest that Madelung deformity has a significant impact on one’s mental and social health. We found that both adults and children experience higher levels of depression and anxiety in comparison with population norms. For adult patients, we found slightly increased depression levels still within normal limits and a mild level of anxiety. For children, depression and anxiety levels were both moderate. Scores varied widely, and both groups included patients with severe levels of depression and anxiety. Regarding social health, adults reported a mildly decreased ability to participate in social roles and activities compared with the general population. Children reported good peer relationships; however, scores again ranged widely, with 53% reporting fair to poor peer relationships. To our knowledge, no other studies on mental or social health in patients with Madelung deformity exist. Previous studies of other congenital upper extremity differences have reported varying results regarding mental and social health outcomes, ranging from no effect to significant impairments.^9,11^ It could be argued that Madelung deformity is associated with higher levels of pain compared with other congenital upper limb differences, in turn resulting in poorer mental and social health.

For both adults and children, decreased physical health appears to be associated with decreased mental and social health levels. Pain and impaired physical functioning have bidirectional associations with depression, anxiety, and social participation.^32,33^ It is plausible that pain and impaired function lead to a vicious circle with decreased levels of physical, mental, and social health, emphasizing the need for treatments improving pain and functioning. Moreover, it also highlights the need for psychological screening and support in both initial work-up and postsurgical follow-up.

The patient perspective is crucial in the treatment of rare congenital anomalies. For most rare diseases, it is unfeasible to develop disease-specific measures or conduct methodologically sound validation studies due to insufficient patient numbers.^15,16^ As demonstrated by Timberlake et al,^13^ social media appears to be a potentially useful tool to study these specific populations; however, an implementation to investigate patient outcomes has not been described. We were able to engage a considerable international audience, compensating the low patient numbers as seen in single-center and even multicenter study designs,^12^ by harnessing social media to assess patient outcomes in a rare population. We believe that selecting an appropriate survey or questionnaire is paramount; PROMIS offers a suitable solution, given that its measurement properties have been thoroughly examined, and it is intended for use in every patient population, including the variety of congenital hand differences.^14^ The possibility of distributing PROMIS with short forms or computer-adaptive tests allows for the measurement of multiple health domains with relatively few questions,^34^ enabling a full assessment of a patient’s health status without being too much of a burden while taking into consideration the importance of material readability.^35^ Moving forward, the relatively low presence of surgeons and academic institutions on social media platforms has been demonstrated, and there is substantial room for improvement both for research and for education.^36^ As clinicians need to gain a full understanding of the burden their patients carry, the combination of our and previous study designs using social media surveys^37^ could serve as a blueprint for assessing the health status of other rare conditions.

The main limitation was that our survey data were obtained from social media communities on Facebook and Instagram, making the methodology prone to self-reporting bias.^38^ In addition, although we limited the survey to participants with “diagnosed” Madelung deformity after a medical assessment, the possibility remained that an undiagnosed or wrongly diagnosed (eg, posttraumatic “Madelung-like” deformity) person would participate. That being said, we considered the probability of obtaining incorrect data to be relatively limited as each entry was checked manually, and questions were added for screening purposes. Finally, we did not distribute our survey on the social media platform Twitter. However, as it has the fewest patient users and minimal engagement, we believe the effect on inclusions to be negligible.^39^ Despite these limitations, to the best of our knowledge, this is the largest study on Madelung deformity outcomes and the first to assess the patient outcomes regarding physical, mental, and social health. We used PROMIS as a self-assessment tool, which has been extensively validated in large populations and proven to be more feasible than other known instruments for patients with congenital hand differences.^40^

In conclusion, Madelung deformity has significant effects on patients’ physical, mental, and social well-being. Even after surgical treatment, the health burden of this congenital hand difference is observed. Using social media, we were able to compensate for Madelung deformity’s rarity by engaging an international audience, demonstrating the feasibility to conduct research through it, and providing a global perspective of the disease entity.

## Supplemental Material

sj-jpg-1-han-10.1177_15589447211054133 – Supplemental material for #MadelungDeformity: Insights Into a Rare Congenital Difference Using Social Media Click here for additional data file. Supplemental material, sj-jpg-1-han-10.1177_15589447211054133 for #MadelungDeformity: Insights Into a Rare Congenital Difference Using Social Media by Abbas Peymani, Max M. Lokhorst, Austin D. Chen, Chantal M.A.M. van der Horst, Bernard T. Lee, Samuel J. Lin and Simon D. Strackee in HAND

sj-pdf-2-han-10.1177_15589447211054133 – Supplemental material for #MadelungDeformity: Insights Into a Rare Congenital Difference Using Social Media Click here for additional data file. Supplemental material, sj-pdf-2-han-10.1177_15589447211054133 for #MadelungDeformity: Insights Into a Rare Congenital Difference Using Social Media by Abbas Peymani, Max M. Lokhorst, Austin D. Chen, Chantal M.A.M. van der Horst, Bernard T. Lee, Samuel J. Lin and Simon D. Strackee in HAND
