# Supplementary figures and images for: #MadelungDeformity: Insights Into a Rare Congenital Difference Using Social Media
Source: Hand (N Y). 2021 Nov 12;18(2 Suppl):24S–31S. doi: 10.1177/15589447211054133 (PMC10052623; doi:10.1177/15589447211054133)

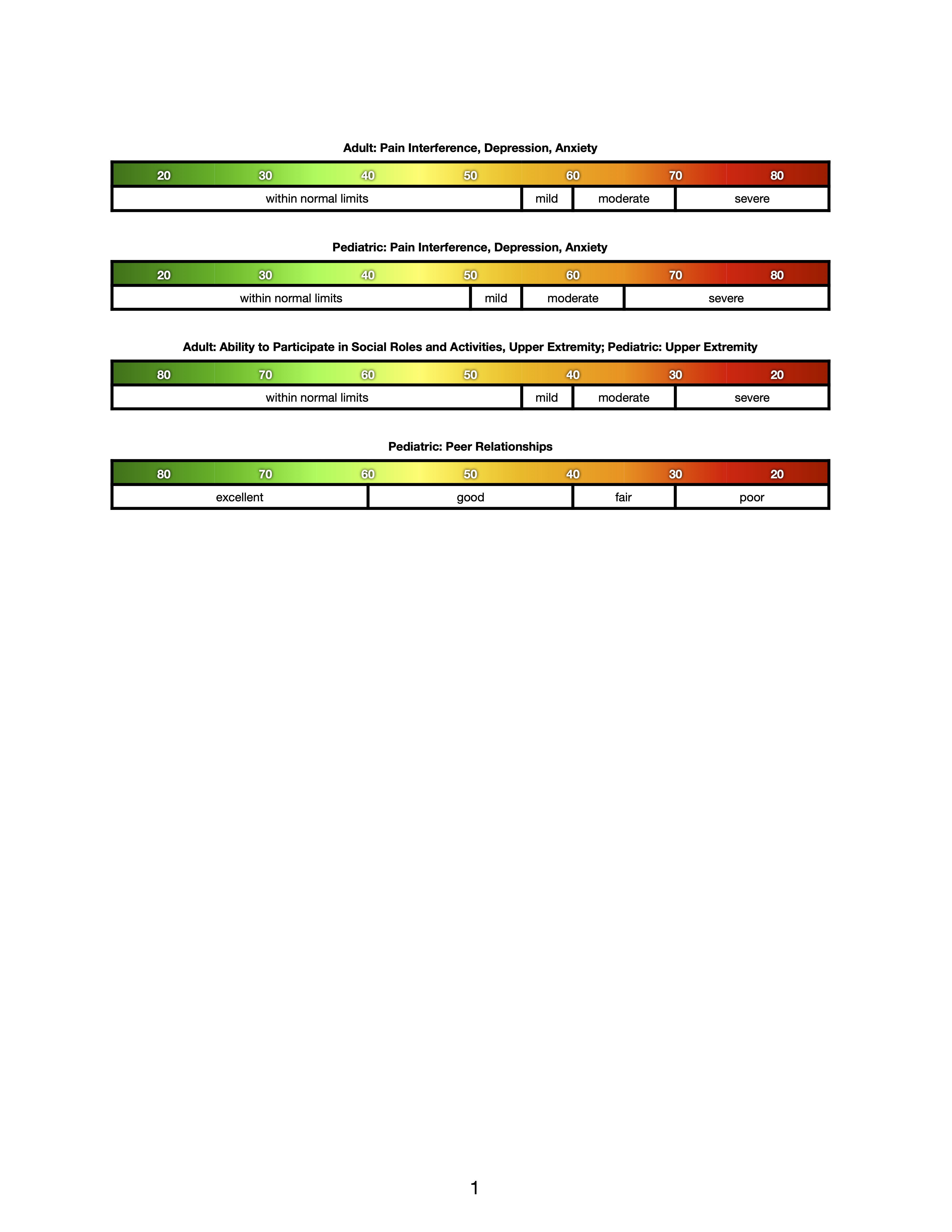

Supplement: sj-jpg-1-han-10.1177_15589447211054133 – Supplemental material for #MadelungDeformity: Insights Into a Rare Congenital Difference Using Social Media [file sj-jpg-1-han-10.1177_15589447211054133.jpg]
