# Supplementary material for: #MadelungDeformity: Insights Into a Rare Congenital Difference Using Social Media
Source: Hand (N Y). 2021 Nov 12;18(2 Suppl):24S–31S. doi: 10.1177/15589447211054133 (PMC10052623; doi:10.1177/15589447211054133)
Supplement: sj-pdf-2-han-10.1177_15589447211054133 – Supplemental material for #MadelungDeformity: Insights Into a Rare Congenital Difference Using Social Media [file sj-pdf-2-han-10.1177_15589447211054133.pdf]

Madelung Deformity Survey

1	**What is your age?**	
2	**What gender were you assigned at birth?**	☐ Female<br>☐ Male<br>☐ Prefer not to say
3	**What is your height?**	
4	**What is your weight? (please add 'pounds' or 'kg')**	
5	**At what age (approximately) were you diagnosed with Madelung deformity?**	
6	**Which hand is your dominant hand?**	☐ Left<br>☐ Right<br>☐ Both (ambidextrous)
7	**Which arm(s) is/are affected?**	☐ Both (left and right)<br>☐ Left only<br>☐ Right only
8	**If you have any genetic conditions, please list below:**	
9	**If you have any other medical conditions, please list below:**	
10	**If you use any medication (including painkillers), please list below:**	
11	**If Madelung deformity occurs in your family, please list below:**<br>*Example: mother, aunt on mother's side, etc.*	
12	**Have you ever had surgery for Madelung deformity?**	☐ Yes<br>☐ No
13	**List all the surgeries you underwent for Madelung deformity. Include your age at surgery, which hand, and the name of the procedure (if you remember):**<br>*Example: 19, left, osteotomy | 24, right, ligament release*	

Question 13 only appears if the answer to Question 12 was ‘Yes’.
